# Pathogenic Role of Immune Cells in Rheumatoid Arthritis: Implications in Clinical Treatment and Biomarker Development

**DOI:** 10.3390/cells7100161

**Published:** 2018-10-09

**Authors:** Hooi-Yeen Yap, Sabrina Zi-Yi Tee, Magdelyn Mei-Theng Wong, Sook-Khuan Chow, Suat-Cheng Peh, Sin-Yeang Teow

**Affiliations:** 1 Department of Medical Sciences, School of Healthcare and Medical Sciences, Sunway University, Jalan Universiti, Bandar Sunway, 47500 Subang Jaya, Selangor Darul Ehsan, Malaysia; 17084351@imail.sunway.edu.my (H.-Y.Y.); sabrinateeziyi@yahoo.com (S.Z.-Y.T.); magdelyn.w@gmail.com (M.M.-T.W.); skhuanchow@gmail.com (S.-K.C.); pehsc@sunway.edu.my (S.-C.P.); 2 Sunway Medical Centre, Jalan Lagoon Selatan, Bandar Sunway, 47500 Subang Jaya, Selangor Darul Ehsan, Malaysia

**Keywords:** rheumatoid arthritis, biomarker, immune cells, B-cells, T-cells, treatment, DMARDs

## Abstract

Rheumatoid arthritis (RA) is a chronic, autoimmune, systemic, inflammatory disorder that affects synovial joints, both small and large joints, in a symmetric pattern. This disorder usually does not directly cause death but significantly reduces the quality of life and life expectancy of patients if left untreated. There is no cure for RA but, patients are usually on long-term disease modifying anti-rheumatic drugs (DMARDs) to suppress the joint inflammation, to minimize joint damage, to preserve joint function, and to keep the disease in remission. RA is strongly associated with various immune cells and each of the cell type contributes differently to the disease pathogenesis. Several types of immunomodulatory molecules mainly cytokines secreted from immune cells mediate pathogenesis of RA, hence complicating the disease treatment and management. There are various treatments for RA depending on the severity of the disease and more importantly, the patient’s response towards the given drugs. Early diagnosis of RA and treatment with (DMARDs) are known to significantly improve the treatment outcome of patients. Sensitive biomarkers are crucial in early detection of disease as well as to monitor the disease activity and progress. This review aims to discuss the pathogenic role of various immune cells and immunological molecules in RA. This review also highlights the importance of understanding the immune cells in treating RA and in exploring novel biomarkers.

## 1. Introduction

Rheumatoid arthritis (RA) is a chronic, autoimmune, systemic disorder that manifests as inflammation of synovial joints, leading to joint destruction and deformity. Although RA is not a fatal disease in general, but the complication associated with RA such as heart diseases and respiratory problems can lead to increased mortality. Common conditions in RA patients are Carpal tunnel syndrome, tendon rupture, cervical myelopathy and joint damage. Other inflammatory complications are Sjögren’s syndrome, pericarditis, pleuritis, scleritis, vasculitis and sexual dysfunction. Due to the long-term disease complication, RA is also associated with a reduced life expectancy. The clinical presentation of rheumatoid arthritis appears between the age of 20 and 40 years old, more commonly in women than in men with a 2–3:1 ratio [1]. In Malaysia, the most common age group that suffers from RA are those in the 30–50. According to the World Health Organization (WHO), within 10 years of onset, at least 50% of patients in developed countries are unable to hold down a full-time job, presumably due to the disability that ensues [1]. The annual incidence of RA in northern Europe and the United States is 0.15–0.6 per 1000, while worldwide 0.5–1.0% of the population is affected [2]. The identified risk factors for the disease are female gender, age, tobacco use, silica exposure and obesity [2].

Since RA is a chronic disease, the treatment mainly focuses on alleviating pain, prevent or limit joint damage, improve or preserve function of the joints and optimize the quality of life [3]. DMARDs are currently the first line of treatment for RA. DMARDs function by modulating various aspects of immune and inflammatory responses that cause clinical manifestations of RA [3]. The most commonly used DMARDs is methotrexate (MTX), followed by others such as hydroxychloroquine, leflunomide, sulfasalazine, azathioprine and cyclosporine. As RA has an insidious course, with more than half of the patients having radiographically visible joint damage within 2 years of onset, it is critical for DMARDs to be administered early [2]. Early treatment is directly associated with decreased progression of synovitis and bone erosions, and significantly improves the prognosis in terms of disability [2]. It is generally advisable for DMARDs to be administered within the first 3 months of the disease for patients with established RA. Since it takes 2–6 months for the conventional DMARDs to reach maximum effect, non-steroidal anti-inflammatory drugs and glucocorticoids can be used in the interim for pain-relief and to reduce inflammation [3]. However, there are substantial side effects of DMARDs that must be taken into consideration. For instances, there is an increased risk of tuberculosis infection associated with use of biological DMARDs such as anti-tumor necrosis factor (TNF) therapy [4,5]. Hence, patients on these therapies are advised to undergo screening for tuberculosis in a timely manner. The use of MTX and leflunomide often causes side effects such as rashes, stomach upset, diarrhea and liver enzymes abnormality. The most common side effects of azathioprine are nausea and vomiting, sometimes with stomach pain and diarrhea whereas kidney damage is the common side effect of cyclosporine.

Depending on the patients’ condition, more specific treatments are being used to treat RA. TNF inhibitors are one of the approved target-specific treatments for RA [6]. In this therapy, TNF is targeted and blocked by the inhibitors to cease the inflammation signaling that causes RA. Similar to DMARDs, anti-TNF therapy has also been associated with increased risk of tuberculosis [7]. On the other hand, T-cells have also been targeted for inhibition to minimize the T-cell-mediated killing of ‘own’ cells which is the main root cause of all autoimmune disorders. Besides that, researchers and pharmaceutical companies are also collectively investigating the possibility of using B-cell and T-cell depletion treatment [8]. However, it should be noted that these biological therapies are more expensive and are usually given to those patients who do not respond to MTX or other conventional DMARDs. Recently, a non-biologic DMARD, Janus kinase (JAK) inhibitors such as ruxolitinib and tofacitinib have been approved in USA. These inhibitors block the activation, mainly phosphorylation of JAK and may subsequently block the intracellular inflammatory process [9]. This group of drugs is used on those who do not respond to MTX alone or MTX in combination with biological therapies as mentioned above [9].

Today, medical practitioners stress the importance of early diagnosis and personalized treatment in preventing or minimizing joint destruction in treating RA [2]. Having this as the treatment strategy for RA and coupled with frequent assessment of disease activity, patients are reported to have improved prognosis [10]. This review paper discusses the role of the host’s immune system, the immune cells and the secretory immunological molecules in the pathogenesis of RA. This review also summarizes current RA treatment targeting immune cells or immunomodulatory molecules as well as the development of immunological biomarkers for RA.

## 2. Pathogenic Role of Immune Cells in Rheumatoid Arthritis

As an autoimmune disorder, immune cells mainly B-cells, T-cells and macrophages play critical roles in RA pathogenesis. These cells can either reside in synovium or circulate in peripheral blood. B-cells secrete physiologically important proteins such as rheumatoid factors (RFs), anti-citrullinated protein antibodies (ACPA) and pro-inflammatory cytokines in supporting RA. B-cells also mediate T-cell activation through expression of costimulatory molecules. In RA, the main function of T-cells is to activate macrophages and fibroblasts and transform them into tissue-destructive cells. Similar to T- and B-cells, activated macrophages produce a variety of cytokines and chemokines to support the inflammation in the joints. This section provides detailed information on how various immune cells contribute to RA pathogenesis.

### 2.1. B-Lymphocytes

B-cells are well known to be an important component in the human adaptive immunity but in the case of RA it also functions as one of the underlying factors of RA onset [11]. Autoreactive B-cells are B-cells that identify host antigens and follow with destruction of such cells or tissues [11]. Autoreactive B-cells are normally eliminated by repairing mechanisms either during the progression from early immature to immature B-cells in the bone marrow, or before the B-cells become mature naïve B-cells [12]. Both of these processes are highly regulated by two immune checkpoints: the central and peripheral B-cell tolerance checkpoints [12]. The central B-cell tolerance checkpoint is controlled by B-cell growth factors that regulate B-cell receptor (BCR) and toll-like receptor (TLR) signaling [13]. In the peripheral B-cell tolerance, it involves extrinsic B-cell factors such as regulatory T-cells (Treg) and serum B-cell activating factor (BAFF) [11].

In RA patients, both checkpoints are usually defective, leading to the large production of autoreactive mature naïve B-cells. As shown in a previous study, untreated RA patients shows a 3.4-fold increase in autoreactive B-cells in the peripheral blood compared to non-RA patients [14]. Such defect can be caused by a mutation in *PTPN22* gene that disrupts the BCR signaling pathway in central B-cell tolerance checkpoint [15]. The impairment of such tolerance checkpoint in RA patients cannot be effectively treated with drugs that reduces inflammation and alleviates other clinical presentations due to the irreversible genetic defect [16]. The impaired peripheral tolerance checkpoint is also evident as shown by the elevated levels of mature naive B-cells that express both polyreactive and human epithelial (HEP-2)-reactive antibodies in RA patients [14]. The peripheral checkpoint dysfunction results in defects in Tregs as well as B-cell resistance to suppression and apoptosis [17,18]. BAFF is increased in the presence of cytokines and chemokines, as well as through TLRs activation in RA patients. Such increase in BAFF expression further prolongs the survival and maturation of autoreactive B-cells, hence sustaining the inflammation and exacerbating the autoimmune conditions [19]. The main culprit of RA, autoreactive B-cells also play role in autoantibody production, T-cell activation and pro-inflammatory cytokine production that ultimately contribute to RA pathogenesis [11]. The underlying mechanisms of autoreactive B-cells targeting host cells remain unclear but the autoantibodies that are associated with RA are well documented and the list continues to expand [11]. The two most studied autoantibody groups are RFs and ACPA [1]. These two autoantibodies are key diagnostic markers that are extremely important in clinical management of RA. Autoreactive B-cells can also act as an antigen presenting cell (APC) in stimulating T-cells maturation and differentiation into memory CD4+ T-cells [20]. This B-cell-dependent T-cell activation is via expression of costimulatory molecules. Local synthesis of cytokines such as TNF-α, IL-6, IL-12, IL-23 and IL-1α due to localized autoreactive B-cells have also been recently reported to act on pathologically relevant cells in RA leading to immune dysfunction, inflammation and bone damage [21]. The bone resorption activity is mediated by osteoclasts (OCs) in which the differentiation and activation require the binding of a cytokine, receptor activator of nuclear factor κB ligand (RANKL) to its receptor, RANK on the osteoclast precursors [22]. The production of RANKL is elevated in the memory B cells from peripheral blood and synovial fluid and tissues of RA patients compared to healthy individuals [23]. The same study also suggested that the B-cells expressing RANKL was highly associated with the OCs differentiation [23].

### 2.2. T-Lymphocytes

In the past decade, extensive studies have been carried out trying to understand the role of T-cells in RA especially the T-cell activation [24]. T-cells can be activated by various cell types including B-cell, macrophages and dendritic cells (DCs). Although the exact role of T-cells in RA remains unclear, there are convincing evidences supporting that CD4+ T-cells contribute significantly to the chronic autoimmune response of RA. During activation of T-cells, CD4+ T-cells interact with human leukocyte antigen (HLA) or major histocompatibility class II (MHC-II) molecules as well as co-stimulating molecules such as CD28 that are expressed on the surface of APC [25]. This interaction then leads to the onset of downstream PI3K signaling pathway leading to the maturation of CD4+ cells [25]. Subsequently, it results in the antigenic activation of naive CD8+ T-cells that promotes inflammation [26]. The role of CD4+ T-cells in RA chronic inflammation is also supported by its association with the particular MHC-II alleles, HLA-DR4 which contain similar amino acid motifs in the third hypervariable region of DRB-chain. This interaction then leads to a more aggressive form of RA [27]. Furthermore, it has been reported that CD4+CD28^null^ correlated with systemic morbidities associated with RA such as vasculitis and acute coronary syndrome [27]. 

In addition to cell-to-cell interaction, current evidences also suggest that CD4+ T-helper (Th) cells mainly contribute to the pathogenesis of RA through the secretion of cytokines and chemokines (will be discussed in Section 3). These molecules are important immune modulators in cell-mediated immunity [24]. Type 1 T-helper (Th1) cells are highly activated in RA and they secrete pro-inflammatory cytokines such as IFN-gamma (IFN-γ), IL-2 and TNF-α [24]. Besides, Th1 cells activate macrophages to act as an APC to present MHC-II molecules to the T-cells [27]. Another type of CD4+ type 2 T-helper (Th2) cells, on the other hand, secrete anti-inflammatory cytokines such as IL-4 and IL-5 and play central roles in B-cell activation and immunoglobulin (Ig) class switching to IgE [28]. Other T-cell subsets such as Th17 and regulatory T (Treg) cells also play central roles in RA pathogenesis [29]. Th17 cells primarily secretes IL-17 that stimulates the production of pro-inflammatory cytokines, chemokines and matrix metalloproteinases (MMPs) [29]. In the past, higher number of Th17 and higher expression level of IL-17 have been consistently detected in RA patients’ serum compared to healthy individuals [30]. Some studies supported that both serum IL-17 and circulating Th-17 cells positively correlate with RA disease activity through disease activity score of 28 joints (DAS28) as well as common markers for RA such as C-reactive protein (CRP) and erythrocyte sedimentation rate (ESR) [30]. Of note, macrophages and mast cells are also main source of IL-17 in addition to Th-17 cells [31]. IL-17 enhances the production of vascular endothelial growth factor-A (VEGF-A), IL-6, IL-8, MMP-1 and MMP-3 in RA synovial fibroblasts [32]. Several studies have shown that IL-17 contributed to pannus growth, osteoclastogenesis and synovial neoangiogenesis [32]. Th-9 cells expressing IL-9 has also been shown to be elevated in the synovial tissues and fluid of RA patients [33]. The Th-9 cells positively correlated with DAS28-ESR of RA patients while synovial IL-9 prolonged the survival of neutrophils, stimulated the production of MMP-9 and facilitated Th17 cell differentiation [34].

On the other hand, CD4+CD25+ Treg cells mainly prevent autoimmunity by suppressing autoreactive lymphocytes mediated by IL-10 and transforming growth factor-beta (TGF-β) [35]. However, the Treg only function specifically in suppressing the proliferation of effector T cells but failed to suppress pro-inflammatory cytokines such as TNF-α and IL-6 produced from activated T-cells and monocytes [36]. The same group also demonstrated that the use of anti-TNF-α, Infliximab, could restore the Treg activity to inhibit the inflammatory cytokine production. Treg cells have been consistently detected in blood and synovial fluid of RA patients [37]. However, the percentage of Treg cells in RA patients compared to healthy individuals is highly controversial. Similarly, contradictory results have also been seen in RA patients when the Treg cell percentage was correlated with multiple clinical characteristics such as DAS28, age, sex, ESR, CRP, RF and disease duration [18,38].

Note worthily, T-cells also play pivotal role in stimulating B-cells responses and antibody production, which consequently contributes to the pathogenesis of RA [39]. T follicular helper (Tfh) cells are the dominant T-cell population that interacts with B-cells usually within the follicles of secondary lymphoid organs (SLOs) and inflamed peripheral tissues [39]. In general, Tfh cells exhibit B-cell-helper phenotype by highly expressing Bcl6, CXCR5, CXCL13, IL-21, PD-1 and ICOS [39,40]. Interestingly, there is a distinct B-cell-helper T-cell population, known as T peripheral helper (Tph) cells, which do not express CXCR5 and only express modest level of Bcl6 in RA synovium. These CXCR5^−^PD-1^hi^ Tph cells once activated, secrete CXCL13 to recruit B cells for IL-21 production and help in B-cell survival, proliferation and maturation [39].

Taken together, multiple T-cells and the respective effector pathways contribute to RA by primarily mediating the chronic inflammatory process. Th-1 cells that specifically secrete pro-inflammatory cytokines were thought to be the main cells causing RA. Following the discovery of other T-cell subtypes and their role in RA such as Th-2, Th-17 and Treg cells as mentioned above, it is clear that RA pathogenesis is much more complicated. This enhanced our understanding in RA and has significant impact on the disease management and treatment.

### 2.3. Macrophages

Macrophages (Mɸ) are consistently found in synovial tissue. Most Mɸ reside within the tissues in a resting state under normal conditions [41]. However, in an inflamed joint, they regulate the secretion of pro-inflammatory cytokines and damaging enzymes that are associated with inflammatory responses and subsequently leading to joint destruction [41]. Other than producing cytokines and enzymes, Mɸ also mediate multiple RA-related biological processes such as recruitment of lymphocytes, cartilage damage, joint erosion, angiogenesis and fibroblast proliferation [41]. Similar to B-cells, Mɸ acts as an APC and is found to highly express HLA-DR and leukocyte adhesion molecules, which allows Mɸ to participate in T-cells activation alongside B-cells [21]. The Mɸ-mediated T-cell activation results in the production of effector T-cells as well as expression of resulting pro-inflammatory mediators such as IL-1α, IL-1β and MMPs which support the pathogenesis of RA [42].

Due to the important roles in RA, targeting macrophages have been reported to turn the disease into remission mainly by inhibiting inflammation and bone erosion [43]. The switchable property of macrophage allows the pro-inflammatory phenotype (M1) to turn into anti-inflammatory phenotype (M2) [43]. This natural transforming ability has been exploited as one of the therapeutic interventions to treat RA. Other therapeutic strategies targeting macrophages include using small interfering RNA (siRNA) [44], anti-TNF [45] and nanosystems [46]. These therapeutic molecules could be potentially developed into anti-rheumatic drugs.

### 2.4. Other Cells

In addition to B-cells, T-cells and macrophages, other immune cells such as mast cell, DCs and natural killer (NK) cells have also been reported to mediate RA pathophysiology. Their roles in RA and potential therapeutic values are tabulated in Table 1. Mast cells reside in synovia and contribute to inflammation followed by RA [47]. In the past, synovial mast cells have been studied to be a potential biomarker for RA [47]. However, the result was not consistent and the mechanism of mast cells in RA remains unknown. Similarly, RA patients have elevated number of activated DCs in their synovial joint tissues [48]. DCs serve as APC and T-cell inducers, hence playing an indispensable role in initiating the joint inflammation as well as maintaining the pro-inflammatory environment in the synovia [48]. In the past decades, studies have been initiated to look into using DCs to benefit anti-rheumatoid therapy [49]. For instance, tolerogenic DCs (tolDCs) have been loaded with antigen to suppress autoimmune responses *in vivo* in RA [50]. The safety and efficiency for RA therapy using tolDCs is currently being evaluated in Phase 1 trials (discussed in Section 4). Last but not least, the pathogenic role of NK cells, another important cell type in innate immune system, has also been reported in RA. CD56+ NK cells were found to overexpress in inflamed joints and produced higher level of IFN-gamma compared to NK cells from peripheral blood [51]. However, the exact mechanism of NK cells remains unknown. Based on current evidences, there is still a big gap in our understanding in RA. Further investigations are warranted to decipher the complex role of multiple immune cells and other relevant constituents in RA pathogenesis.

## 3. Role of Immune-Related Secretory Molecules in Rheumatoid Arthritis

### 3.1. Cytokines

Pro-inflammatory cytokines play pivotal roles in the pathogenesis of RA [52]. Cytokines are proteins that function as mediators in cell signaling, the term ‘cytokine’ comprises of monokines, lymphokines, ILs, IFNs, colony stimulating factors (CSFs) and chemokines [53]. In the early pathogenesis of RA, the predominant cytokines are IL-13, IL-14 and IL-15 that are secreted from T-cells and stromal cells [54]. These cytokines result in the inflammatory response and contribute to the chronic inflammation. Other cytokines such as IL-6 and tumor necrosis factor alpha (TNF-α) have also been reported to promote RA [55]. The pro-inflammatory cytokines such as TNF- α, IL-1 and IL-17 usually outweigh the protective effects of the anti-inflammatory cytokines such as IL-4, IL-10 and IL-13, which then result in the cytokine-mediated inflammation [56]. In RA, the B-cells and macrophages which are the APCs present arthritis-associated antigens to T-cells and activate the signaling cascades to secrete cytokines [57]. These cytokines subsequently stimulate the activation of both chondrocytes and osteoclasts and produce MMPs that degrade the matrix of articular cartilage leading to bone resorption in RA [56].

Generally, the production of cytokines is initiated by the activated CD4+ T-cells [56]. Other cytokine-producing cells are NK cells, neutrophils, macrophages, monocytes, fibroblasts and mast cells [56]. The CD4+ TH17 cells mainly produce a majority of the ILs such as IL-17, IL-21, IL-22 and IL-23 that contribute to bone erosion due to synovial inflammation [56]. IL-6 is responsible for acute phase responses including anemia, cognitive dysfunction, inactivation of leukocytes and the production of autoantibodies leading to lipid metabolism dysregulation [55]. The IL-17 has been shown to stimulate the production of MMP1 and MMP3, TNF-α, IL-6, IL-8 and other pro-inflammatory cytokines, thereby increasing infiltration of immune cells into the synovium [56]. TNF-α stimulates the proliferation and differentiation of B-lymphocytes, T-lymphocytes and NK cells, as well as the production of other pro-inflammatory cytokines such as IL-1, IL-6 and other mediators causing tissue destruction [56]. TNF-α also causes the expression of adhesion molecules of the endothelial cells and suppresses the Tregs, hence stimulating angiogenesis and the sensation of pain [55].

### 3.2. Antibodies

Autoantibodies play significant roles in the pathogenesis of various autoimmune diseases including RA [58]. When immune dysregulation occurs, the body’s antibodies attack their own antigens. Several antibodies have been consistently found in RA patients including RF, ACPA and regulatory rheumatoid factor (regRF) [59]. RF is an autoantibody that targets the Fc portion of immunoglobulin G [60]. This autoantibody is routinely screened in diagnostics laboratories for RA patient diagnosis [59]. It is important to note that not all cases of RA contain the RF, yet those with RF positive tend to manifest with bone erosion and poorer prognosis [60]. Furthermore, RF is not specific for RA as it has been found in patients with other autoimmune diseases and a variety of infectious diseases [61]. Meanwhile, 10–30% of elderly individuals and 3–5% of the healthy population also express RF in their blood [62], this may cause false positive result, Hence, more sensitive and specific diagnostic marker for RA is always in need. Contradictorily, it has been reported that the regRF could resist disease progression in RA and in fact, promote remission in autoimmune diseases [63]. Although regRF is not present in its native IgG form, it is possible to induce its antigenic determinants in the hinge region of Fc fragments of homologous IgG, making it a potential target for RA therapy [63]. Further investigation is also required to evaluate its potential as a diagnostics marker. Another antibody that is highly pertinent in RA is ACPA (also known as anti-CCP). ACPA is a family of antibodies with overlapping specificities [64]. These antibodies recognize a range of citrullinated proteins such as filaggrin, fibrinogen, vimentin, collagen II, enolase and histones [65]. Similar to RF, ACPA level in the blood is also routinely screened as a diagnostic test for RA [66].

In the past few years, more studies have been conducted on an antibody against carbamylated antigens (known as anti-CarP antibodies). Anti-CarP antibodies was found to correlate with the patients’ joint erosion score but not ACPA [67]. The same study also showed that anti-CarP antibodies correlated with DAS28 when combined with ACPA but not individually [67]. Similarly, Kumar et al. demonstrated that there was a weak negative correlation between anti-CarP and DAS28 and there was no significant correlation with RF [68]. In addition, Shi et al. showed that anti-CarP antibodies have 44% sensitivity and 89% specificity for RA as compared to the ACPA that has 54% sensitivity and 96% specificity [69]. Similar study also demonstrated that anti-CarP antibodies occur in almost all forms of early arthritis such as reactive arthritis and psoriatic arthritis [69]. 

Other antibodies that are also known to associate with RA are anti-K8, anti-p68 and anti-Sa antibodies [70]. Keratin is a functionally diverse intermediate filament which is widely distributed in the human body [70]. K8 is one of the keratin types [70]. The antibody reactive against K8 (anti-K8 antibody) has been demonstrated in about 68% of Han Chinese-originated RA patients [66]. On the other hand, BiP autoantibodies (heavy chain binding protein), formerly known as anti-p68, have been found in 64% of RA patients [71]. Another antibody, anti-Sa antibody which has 92–99% specificity can also be detected from human endothelial cells including RA hypertrophied synovium (also called pannus tissue) [71]. In contrast, a study reported that the anti-Sa antibodies are citrullinated vimentin in which the citrulline moiety acts as autoantigenic hapten and its sensitivity is only ranged from 30–40% [71]. Other RA-associated antibodies are anti-RA33, anti-calpastatin, anti-neutrophil cytoplasmic antibodies (ANCA), antibodies to nuclear antigens (ANA), anti-collagen type II, anti-fibronectin and anti-GPI [70]. However, most of these autoantibodies are not RA-specific as they are also readily found in other autoimmune diseases such as systemic lupus erythematosus (SLE) and osteoarthritis [71].

### 3.3. Other Rheumatoid Arthritis-Associated Soluble Mediators

In addition to the cytokines and antibodies that play important immune-regulatory roles in RA, other soluble mediators such as synovium markers [72], cartilage markers [73], vascular markers [74] and bone markers [75] have also been identified to contribute to RA progression. One of the synovium markers is serum hyaluronan (HA), a natural polysaccharide found in the body’s extracellular matrix (ECM) [65]. HA binds to the CD44 receptors which are overexpressed in the synovial tissue of RA patients. These expression is mediated by the synovial lymphocytes, macrophages and fibroblasts at the inflamed joints [76]. Of note, higher expression of CD44 is found in the inflamed joints of RA patients compared to other conditions such as osteoarthritis (OA) and joint trauma [76]. Another synovium marker is MMPs including MMP-1, MMP-2, MMP-3 and MMP-9 [72,77]. MMP-1 mainly degrades collagen in the ECM while MMP-2, MMP-3 and MMP-9 degrade non-collagen matrix components of the joints [77]. The level of MMPs has been reported to elevate in RA patients [77]. These enzymes mainly function in ECM cleavage and produce bioactive by-products as the result of the processes [78]. MMPs also play key roles in bone remodeling, including osteoblast/osteocyte differentiation, bone formation, solubilization of the osteoid and osteoclast recruitment and migration [78].

**Figure 1:**
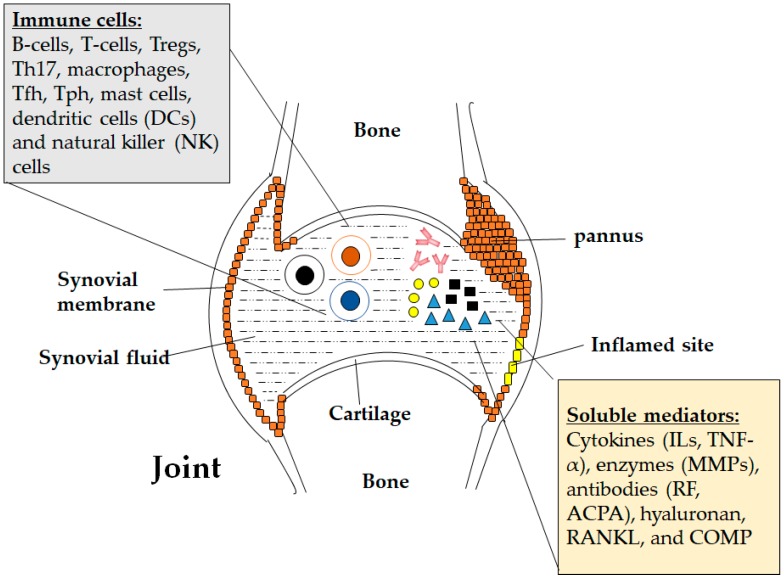
Host immune cells and soluble mediators in rheumatoid arthritis pathogenesis.

Cartilage-specific markers such as cartilage oligomeric matrix protein (COMP) have also been found to contribute to RA [73]. COMP is a homopentamer of 524 kDa size that is mostly found in cartilage, followed by other sites such as tendons, meniscus, ligaments and synovium [79]. Various proteases have been found to degrade the ECM of articular cartilage and release several protein fragments as by-products, COMP is one of them [79]. Similar to CD44, higher level of COMP is found in RA patients compared to OA patients [11]. When the COMP level was compared within the category of RA patients with slow and rapid progression of joint damage, COMP is distinguishably higher in those with severed joint destruction [80]. This characteristic of COMP makes it useful for identifying patients that are at high risk for joint destruction and can be potently used as a biomarker to monitor cartilage degradation [79]. Other soluble mediators are bone markers such as bone sialoprotein and cross-linked carboxyterminal telopeptides of type I collagen (ICTP) [75] and vascular markers such as serum vascular endothelial factor [74]. All of these soluble factors contribute differently to RA and their potentials to be developed into diagnostic and predictive markers are being investigated. Figure 1 shows the immune cells and soluble mediators that contribute to the pathogenesis of RA.

## 4. Targeting Immunological Components for Rheumatoid Arthritis Treatment

Due to the indispensable roles of immunological components in RA pathogenesis, they have been targeted for clinical therapies development. These RA-specific targets comprise of immune or non-immune cells, cellular receptors and soluble factors. The cell-based therapies include B-cells and T-cell depletion [80]. The RA therapies target cellular receptors such as IL-6 receptors [81] and CD20 [82] as well as membrane-bound and soluble factors (mainly cytokines) such as GM-CSF, BAFF and TNF-α [83,84]. This section discusses the current therapies that are directed against the human immune system to treat RA and the potential treatment for RA which are currently under investigational clinical trials.

### 4.1. FDA-Approved Therapies Against Rheumatoid Arthritis

Disease-modifying anti-rheumatic drugs (DMARDs) are standard medication for RA patients, methotrexate is one of the commonly used drug to suppress autoimmune response but its side effects is notorious [85]. Over the years, more advanced and aggressive DMARDs treatment have been used alone and in combination with several treatments specifically targeting immune cells and the related immune-modulators [86]. These treatment is also known as biologics. Amongst all, anti-TNF-α treatment is the most popular group in treating RA patients [87]. Clinically used drugs that are acting on TNF-α include Infliximab [88], Adalimumab [89], Etanercept [90], Certolizumab [91] and Golimumab [92]. The anti-TNF mechanisms of these drugs are tabulated in Table 2. Other drugs targeting other immune modulators such as CD20 on the B cells (Rituximab) [93] and IL-6 receptors (Tocilizumab) [94] are also being used to treat RA patients. Rituximab is usually used on patients that do not respond well to DMARDs including the anti-TNF therapy. The most recent FDA-approved drug for RA was sarilumab, which is an antagonist for IL-6 receptor [95]. Many other potential therapies targeting immune cells and the related components such as cytokines, growth factors and cell receptors are currently in clinical trials (will be discussed in next section). It should be highlighted that other anti-RA drugs targeting Janus kinase (JAK) such as tofacitinib (approved in 2012) [96] and baricitinib (approved in 2018) [97] inhibit the phosphorylation of JAK and may play role in inhibiting inflammatory processes.

### 4.2. Potential Rheumatoid Arthritis Therapies in Clinical Trials

Various treatments targeting immune cells and related components are currently being evaluated in clinical trials (Table 3). As stated earlier, RA treatment adopting tolDCs is one of the examples [50]. This therapy specifically targets the pathogenic autoimmune response while leaving the protective immunity intact. The safety and efficiency of this immunotherapy is currently under assessment. A nanoparticle-based immunotherapy, DEN-181 is currently in Phase I trial in Brisbane, Australia [99]. Unlike other anti-rheumatic drugs, DEN-181 targets the cause of RA by regulating activated immune cells which cause inflammation, rather than treating the symptoms. Another drug, AMG-592 that targets inflammation in RA is also in Phase 1 trial [100]. AMG-592 is a fusion protein of IL2-mutein and human Fc. This drug was designed to improve Treg selectivity and half-life compared to recombinant IL-2. Mavrilimumab targeting BAFF is currently in Phase 2 trial [101]. Another Phase 3 trial evaluating BAFF-targeting drug, Tabalumab/LY2127399 has been terminated due to lack of efficacy but not the safety concerns [102]. Namilumab (also known as AMG-203 or MT203) is a monoclonal antibody targeting GM-CSF ligand and had completed Phase 2 trial in 2016 [103]. Similarly, monoclonal antibodies targeting GM-CSF such as GSK3196165 [104] and MORAb-022 [105] are being evaluated in clinical trials (Table 3). Meanwhile, Phase 2 trial using KB003 antibody targeting GM-CSF has been terminated upon completion of safety run-in because of the development program refocus [106]. 

## 5. Immune Cell Related Biomarker and Vaccine Development for Rheumatoid Arthritis

### 5.1. Disease Activity and Diagnostic Markers

Currently, the diagnostic markers for RA stated in the ACR/EULAR 2010 criteria are RF and anti-CCP antibodies [108,109]. Anti-mutated citrullinated vimentin (anti-MCV) that produced similar results when used along with anti-CCP antibodies. This can also be used as a second-line biomarkers [110]. Another diagnostic marker that can be used to improve RA diagnostics is the 14-3-3 eta protein. This protein has been previously tested and showed an increase in percent of diagnosis success rate, from 72% (RF and anti-CCP without 14-3-3 eta protein) to 78% (with 14-3-3 eta protein) [111]. As RA is an inflammatory condition, both the erythrocyte sedimentation rate (ESR) and C-reactive protein (CRP) are significant indicators of the disease severity. According to the 1987 ACR classification criteria, active disease is defined as having a disease activity score in 28 joints based on an ESR of >5.1 at baseline [112]. When compared to ESR, CRP was found to be a better measurement of inflammation and was a more accurate representation of the inflammatory component provided by both the tender joint count and the swollen joint count [113]. In order to monitor the disease activity in patient, doctors usually use a couple of guideline disease evaluation score such as DAS28, simple disease activity index (SDAI) and clinical disease activity index (CDAI) [113]. However, the negative effect of using such score is because some of the criteria are subjective, as well as patients still suffer from RA although no inflammation was observed in the patients. For better disease evaluation, another form of scoring was developed by determining multiple biomarkers in patient. This evaluation score is called the multi-biomarkers disease activity test (MBDA), which determine the disease activity via biomarkers such as CRP, VCAM-1, IL-6, anti-MMP1 as well as EGF and VEGF-A, which give a better RA evaluation compared to the other test [114]. Of note, it was reported that the Vectra DA test, a RA diagnostic test that measures 12 biomarkers in combination including VCAM-1, EGF, VEGF-A, IL-6, MMP-1, MMP-3, TNF-R1, YKL-40, Leptin, Resistin, SAA (serum amyloid) and CRP produced more accurate results in measuring the disease activity of the patients who are receiving RA treatment [115,116].

### 5.2. Predictive Biomarkers for Rheumatoid Arthritis Treatment

As RA treatment advances, the need of a specific and effective treatment is required. The introduction of biological treatment allows doctors to better manage RA and the increase in understanding of RA allow more specific treatment to be developed. However, studies have reported that some of the patients are non-respondent when treated with biological drugs [117]. In order to achieve a better use of biological drugs as well as reduce the waste of treating non-respondent patients, biomarkers are used to determine the best line of treatment for patient. Such biomarkers as well as the potential effective therapy to be used are listed in Table 4.

### 5.3. Vaccine Development for Rheumatoid Arthritis

Despite the advancement of technologies and understanding about the disease, there is still no cure and no ‘preventive’ measures for RA. Efforts are ongoing to develop a prophylactic or therapeutic vaccine for RA which prevents the disease and the later, improve the therapeutic outcome of disease, respectively. So far, the most prominent RA prophylactic vaccine comprised of autologous DCs that had been modified by NF-κβ inhibitor and exposed to ACPA (designated as Rheumavax) before injecting back into patients. In 2015, the phase I trial using Rheumavax was completed and produced promising results with a few adverse events [124]. However, there is no follow up since then.

Pro-inflammatory cytokines play substantial role in RA pathogenesis. Hence, cytokines have been targeted for therapeutic vaccine development. These cytokines include macrophage inhibitory protein (MIF) [125], IL-2 receptor subunit [125], IL-23 [126], RANKL [127,128], rBAFF [129] and VEGF-B [130]. Most of these experimental vaccination strategies have advanced into pre-clinical animal studies to evaluate their feasibility in human trials [131]. Among these therapeutic vaccines, only denosumab targeting RANKL has completed Phase II trials on RA patients [127,128]. The trial results suggest that denosumab is efficient in both systemic and articular bone loss in RA with limited side effects and the combination with anti-TNF and MTX could enhance the RA treatment outcomes [132].

There have been numerous vaccination strategies that are targeting collagens or the related peptides [133]. Substantial animal studies have been conducted on these collagen-targeting therapeutic vaccines including anti-collagen [134], CTAIR7K-COL-DD [135], CEL-2000 [136] and GalOK264/Aq [137]. Note worthily, CEL-2000 was developed using Ligand Epitope Antigen Presentation System (LEAPS) technology which is a hetero-conjugate containing immune cell binding ligand (ICBL) attached to the antigenic peptide [131,133]. LEAPS has also been used to develop antigen-specific T-cell modulating vaccines such as CTAIR7K-COL-DD [135] and gal-CII259-273 [137]. Furthermore, it has also been reported that *Salmonella* vector with colonization factor antigen-I [138] and *Escherichia coli* enterotoxin B heat labile [139] could potentially be developed into therapeutic vaccines for RA.

## 6. Conclusions

With the enhanced understanding of disease and advancement of therapy, the medical management of RA has significantly been improved in the past decades. The therapeutic objective of RA has been shifted from relieving the disease symptoms to arresting the disease processes. In addition to type of RA treatment, early detection of disease, method of disease measurement, disease classification and the criteria for remission play decisive roles in improving the patient’s treatment outcomes. Immune cells particularly B- and T-lymphocytes are key components involved in each of the aspect as abovementioned. Current evidences suggest that these immune cells can be targeted as one of the therapeutic approaches for RA such as T-cell and B-cell depletion and tolDCs treatments. Further investigations are warranted for in-depth understanding of the functions of immune cells in RA. This shall ultimately lead to a more effective therapeutic modality and improve the life quality of the RA patients.

## Figures and Tables

**Table 1:** Contribution of immune cells to Rheumatoid Arthritis (RA) and their potentials as therapeutic targets.

Cell Type	Subtype	Pathogenic Roles	References
B-cells	-	Antibody producer, APC, T-cell activation and cytokine-producer such as IL-4 and IL-10	[11,12,13]
T-cells	Th-1	Cytokine producer, macrophage activation	[28]
	Th-2	Cytokine producer, B-cell activation, promote Ig class switching to IgE	[28]
	Th-17	Cytokine producer, MMP stimulation, promote pannus growth, neoangiogenesis and osteoclastogenesis	[29,30,31,32]
	Treg	Suppress autoreactive lymphocytes	[35,36,37]
Macrophage	-	APC, T-cell activation, cytokine producer, promote angiogenesis and fibroblast proliferation	[18,41,42]
Mast cells	-	Pro-inflammatory cytokines producer	[47]
Dendritic cells	-	APC and T-cell activation	[48]
Natural killer (NK) cells	-	Pro-inflammatory cytokines producer	[51]

**Table 2:** Current Food and Drug Administration (FDA)-approved drugs targeting immune responses for the treatment of rheumatoid arthritis.

Drug Name	Target and Action	FDA Approval Year	Reference
Etanercept	Dimeric human TNF receptor targeting TNF	1998	[90]
Infliximab	Monoclonal antibody targeting human TNFα	2002	[88]
Adalimumab	Recombinant monoclonal antibody targeting TNF	2002	[89]
Abatacept	Recombinant fusion protein targeting T-lymphocytes activation	2005	[98]
Rituximab	Chimeric monoclonal antibody that targets CD20 molecules of B-cells	2006	[93]
Certolizumab	Humanized and pegylated anti-TNFα inhibitor	2009	[91]
Golimumab	Humanized monoclonal antibody targeting TNFα	2009	[92]
Tocilizumab	Humanized monoclonal antibody that targets IL-6 receptors and blocks signaling	2010	[94]
Sarilumab	IL-6 receptor antagonist	2017	[95]

**Table 3:** Potential drugs for the treatment of rheumatoid arthritis.

Name	Target and Mechanism	Stage	Trial Period	Reference
AMG 592	Improve Treg selectivity	Phase 2	2018–2020	[100]
DEN-181 ^1^	Regulate T-lymphocytes	Phase 1	2018	[99]
Mavrilimumab	BAFF	Phase 2	2013–2015	[101]
Namilumab (MT203)	GM-CSF ligand	Phase 2	2015–2016	[103]
Lenzilumab/KB003	GM-CSF	Phase 2 *(terminated)*	2010–2012	[106]
Tabalumab (LY2127399)	BAFF	Phase 3 *(terminated)*	2011–2014	[102,107]
GSK3196165 (MOR103)	GM-CSF	Phase 2	2015–2017	[104]
MORAb-022	GM-CSF	Phase 1	2013–2014	[105]
AutoDECRA	Inhibit inflammation	Phase 1	2012–2013	[50]

^1^ Not listed in US clinical trials, only in Australia.

**Table 4:** Potential predictive biomarkers for rheumatoid arthritis treatment.

Biomarkers	Presence/Absence	Medication/Drug	References
Anti-CCP	Present	Rituximab	[118,119]
Anti-MCV	Present	Rituximab	[119]
14-3-3 eta	Absent or low level	Tocilizumab, Anti-TNF drugs	[120]
Cartilage oligometic matrix protein (COMP)	Absent or low level	Adalimumab	[121]
Calprotectin	Present	Adalimumab, Infliximab, Rituximab	[122]
Survivin	Absent or low level	Infliximab	[123]

## References

[1] Chronic Rheumatic Conditions. http://www.who.int/chp/topics/rheumatic/en/.

[2] Garneau E., Ferri F.F. (2018). Rheumatoid arthritis. Ferri’s Clinical Advisor.

[3] Kayanaugh A., Grevich S.C., Kellerman R.D., Bope E.T. (2018). Rheumatoid arthritis. Conn’s Current Therapy.

[4] Brassard P., Kezouh A., Suissa S. (2006). Antirheumatic drugs and the risk of tuberculosis. Clin. Inf. Dis..

[5] Navarra S.V., Tang B., Lu L., Lin H.Y., Mok C.C., Asavatanabodee P., Suwannalai P., Hussein H., Rahman M.U. (2014). Risk of tuberculosis with anti-tumor necrosis factor-α therapy: Substantially higher number of patients at risk in Asia. Int. J. Rheum. Dis..

[6] Mewar D., Wilson A.G. (2011). Treatment of rheumatoid arthritis with tumour necrosis factor inhibitors. Br. J. Pharmacol..

[7] Dixon W.G., Hyrich K.L., Watson K.D., Lunt M., Galloway J., Ustianowski A., Symmons D.P., BSRBR Control Centre Consortium, BSR Biologics Register (2010). Drug-specific risk of tuberculosis in patients with rheumatoid arthritis treated with anti-TNF therapy: Results from the British Society for Rheumatology Biologics Register (BSRBR). Ann. Rheum. Dis..

[8] Rheumatoid Arthritis Treatment. http://www.hopkinsarthritis.org/arthritis-info/rheumatoid-arthritis/ra-treatment/.

[9] Semerano L., Decker P., Clavel G., Boissier M.C. (2016). Developments with investigational Janus kinase inhibitors for rheumatoid arthritis. Expert Opin. Investig. Drugs.

[10] Monti S., Montecucco C., Bugatti S., Caporali R. (2015). Rheumatoid arthritis treatment: The earlier the better to prevent joint damage. RMD Open.

[11] Bugatti S., Vitolo B., Caporali R., Montecucco C., Manzo A. (2014). B cells in rheumatoid arthritis: From pathogenic players to disease biomarkers. BioMed Res. Int..

[12] Giltiay N.V., Chappell C.P., Clark E.A. (2012). B-cell selection and the development of autoantibodies. Arthritis Res. Ther..

[13] Browne E.P. (2012). Regulation of B-cell responses by Toll-like receptors. Immunology.

[14] Samuels J., Ng Y.S., Coupillaud C., Paget D., Meffre E. (2005). Impaired early B cell tolerance in patients with rheumatoid arthritis. J. Exp. Med..

[15] Meffre E. (2011). The establishment of early B cell tolerance in humans: Lessons from primary immunodeficiency diseases. Ann. N. Y. Acad. Sci..

[16] Menard L., Samuels J., Ng Y.S., Meffre E. (2011). Inflammation-independent defective early B cell tolerance checkpoints in rheumatoid arthritis. Arthritis Rheum..

[17] Ehrenstein M.R., Evans J.G., Singh A., Moore S., Warnes G., Isenberg D.A., Mauri C. (2004). Compromised function of regulatory T cells in rheumatoid arthritis and reversal by anti-TNFalpha therapy. J. Exp. Med..

[18] Rapetti L., Chabele K.M., Evans C.M., Ehrenstein M.R. (2015). B cell resistance to Fas-mediated apoptosis contributes to their ineffective control by regulatory T cells in rheumatoid arthritis. Ann. Rheum. Dis..

[19] Mackay F., Schneider P. (2009). Cracking the BAFF code. Nat. Rev. Immunol..

[20] Aarvak T., Natvig J.B. (2011). Cell-cell interactions in synovitis: Antigen presenting cells and T cell interaction in rheumatoid arthritis. Arthritis Res..

[21] Schlegel P.M., Steiert I., Kötter I., Müller C.A. (2013). B cell contribute to heterogeneity of IL-17 producing cells in rheumatoid arthritis and healthy controls. PLoS ONE.

[22] Nakagawa N., Kinosaki M., Yamaguchi K., Shima N., Yasuda H., Yano K., Morinaga T., Higashio K. (1998). RANK is the essential signaling receptor for osteoclast differentiation factor in osteoclastogenesis. Biochem. Biophys. Res. Commun..

[23] Cope A.P., Schulze-Koops H., Aringer M. (2007). The central role of T cells in rheumatoid arthritis. Clin. Exp. Rheumatol..

[24] Meednu N., Zhang H., Owen T., Sun W., Wang V., Cistrone C., Rangel-Moreno J., Xing L., Anolik J.H. (2016). Production of RANKL by memory B cells: A link between B cells and bone erosion in rheumatoid arthritis. Arthritis Rheumatol..

[25] Podojil J.R., Miller S.D. (2009). Molecular mechanisms of T cell receptor and costimulatory molecule ligation/blockade in autoimmune disease therapy. Immunol. Rev..

[26] Williams M.A., Bevan M.J. (2007). Effector and memory CTL differentiation. Annu. Rev. Immunol..

[27] Cope A.P. (2008). T cells in rheumatoid arthritis. Arthritis Res. Ther..

[28] Schulze-Koops H., Kalden J.R. (2001). The balance of Th1/Th2 cytokines in rheumatoid arthritis. Best Pract. Res. Clin. Rheumatol..

[29] Alunno A., Manetti M., Caterbi S., Ibba-Manneschi L., Bistoni O., Bartoloni E., Valentini V., Terenzi R., Gerli R. (2015). Altered immunoregulation in rheumatoid arthritis: The role of regulatory T cells and proinflammatory Th17 cells and therapeutic implications. Mediat. Inflamm..

[30] Al-Saadany H.M., Hussein M.S., Gaber R.A., Zaytoun H.A. (2016). Th-17 cells and serum IL-17 in rheumatoid arthritis patients: Correlation with disease activity and severity. Egypt. Rheumatol..

[31] Suurmond J., Dorjée A.L., Boon M.R., Knol E.F., Huizinga T.W., Toes R.E., Schuerwegh A.J. (2011). Mast cells are the main interleukin 17-positive cells in anticitrullinated protein antibody-positive and -negative rheumatoid arthritis and osteoarthritis synovium. Arthritis Res. Ther..

[32] Gaffen S.L. (2009). Role of IL-17 in the pathogenesis of rheumatoid arthritis. Curr. Rheumatol. Rep..

[33] Ciccia F., Guggino G., Rizzo A., Manzo A., Vitolo B., La Manna M.P., Giardina G., Sireci G., Dieli F., Montecucco C.M., Alessandro R., Triolo G. (2015). Potential involvement of IL-9 and Th9 cells in the pathogenesis of rheumatoid arthritis. Rheumatology (Oxford).

[34] Chowdhury K., Kumar U., Das S., Chaudhuri J., Kumar P., Kanjilal M., Ghosh P., Sircar G., Basyal R.K., Kanga U., Bandyopadhaya S., Mitra D.K. (2018). Synovial IL-9 facilitates neutrophil survival, function and differentiation of Th17 cells in rheumatoid arthritis. Arthritis Res. Ther..

[35] Cooles F.A., Isaacs J.D., Anderson A.E. (2013). Treg cells in rheumatoid arthritis: An update. Curr. Rheumatol. Rep..

[36] Boissier M.C., Assier E., Biton J., Benys A., Falgarone G., Bessis N. (2009). Regulatory T cells (Treg) in rheumatoid arthritis. Jt. Bone Spine.

[37] Morita T., Shima Y., Wing J.B., Sakaguchi S., Ogata A., Kumanogoh A. (2016). The proportion of regulatory T cells in patients with rheumatoid arthritis: A meta-analysis. PLoS ONE.

[38] Al-Zifzaf D.S., El Bakry S.A., Mamdouh R., Shawarby L.A., Ghaffar A.Y.A., Amer H.A., Alim A.A., Sakr H.M., Rahman R.A. (2015). FoxP3+T regulatory cells in rheumatoid arthritis and the imbalance of the Treg/TH17 cytokine axis. Egypt. Rheumatol..

[39] Rao D.A. (2018). T cells that help B cells in chronically inflamed tissues. Front. Immunol..

[40] Rao D.A., Gurish M.F., Marshall J.L., Slowikowski K., Fonseka C.Y., Liu Y., Donlin L.T., Henderson L.A., Wei K., Mizoguchi F. (2017). Pathologically expanded peripheral T helper cell subset drives B cells in rheumatoid arthritis. Nature.

[41] Kinne R.W., Bräuer R., Stuhlmüller B., Palombo-Kinne E., Burmester G.R. (2000). Macrophages in rheumatoid arthritis. Arthritis Res..

[42] Bondeson J., Wainwright S.D., Lauder S., Amos N., Hughes C.E. (2006). The role of synovial macrophages and macrophage-produced cytokines in driving aggrecanases, matrix metalloproteinases, and other destructive and inflammatory responses in osteoarthritis. Arthritis Res. Ther..

[43] Davignon J.L., Hayder M., Baron M., Boyer J.F., Constantin A., Apparailly F., Poupot R., Cantagrel A. (2013). Targeting monocytes/macrophages in the treatment of rheumatoid arthritis. Rheumatology (Oxford).

[44] Kim S.S., Ye C., Kumar P., Chiu I., Subramanya S., Wu H., Shankar P., Manjunath N. (2010). Targeted delivery of siRNA to macrophages for anti-inflammatory treatment. Mol. Ther..

[45] Onuora S. (2018). Experimental arthritis: Anti-TNF kills the macrophage response. Nat. Rev. Rheumatol..

[46] Pham C.T. (2011). Nanotherapeutic approaches for the treatment of rheumatoid arthritis. Wiley Interdiscip. Rev. Nanomed. Nanobiotechnol..

[47] Rivellese F., Nerviani A., Rossi F.W., Marone G., Matucci-Cerinic M., de Paulis A., Pitzalis C. (2017). Mast cells in rheumatoid arthritis: Friends or foes?. Autoimmun. Rev..

[48] Yu M.B., Langridge W.H.R. (2017). The function of myeloid dendritic cells in rheumatoid arthritis. Rheumatol. Int..

[49] Khan S., Greenberg J.D., Bhardwaj N. (2009). Dendritic cells as targets for therapy in rheumatoid arthritis. Nat. Rev. Rheumatol..

[50] Hilkens C.M., Isaacs J.D. (2013). Tolerogenic dendritic cell therapy for rheumatoid arthritis: Where are we now?. Clin. Exp. Immunol..

[51] Shegarfi H., Naddafi F., Mirshafiey A. (2012). Natural killer cells and their role in rheumatoid arthritis: Friend or foe?. Sci. World J..

[52] Feldmann M., Brennan F.M., Maini R.N. (1996). Role of cytokines in rheumatoid arthritis. Annu. Rev. Immunol..

[53] Brzustewicz E., Bryl E. (2015). The role of cytokines in the pathogenesis of rheumatoid arthritis—Practical and potential application of cytokines as biomarkers and targets of personalized therapy. Cytokine.

[54] Burska A., Boissinot M., Ponchel F. (2014). Cytokines as Biomarkers in Rheumatoid Arthritis. Med. Inflamm..

[55] Alam J., Jantan I., Bukhari S.N.A. (2017). Rheumatoid arthritis: Recent advances on its etiology, role of cytokines and pharmacotherapy. Biomed. Pharmacother..

[56] Mateen S., Zafar A., Moin S., Khan A.Q., Zubair S. (2016). Understanding the role of cytokines in the pathogenesis of rheumatoid arthritis. Clin. Chim. Acta.

[57] Choy E. (2012). Understanding the dynamics: Pathways involved in the pathogenesis of rheumatoid arthritis. Rheumatology (Oxford).

[58] Griesmacher A., Peichl P. (2001). Autoantibodies associated with rheumatic diseases. Clin. Chem. Lab. Med..

[59] Song Y.W., Kang E.H. (2010). Autoantibodies in rheumatoid arthritis: Rheumatoid factors and anticitrullinated protein antibodies. QJM-Int. J. Med..

[60] Tseng W.Y., Jan Wu Y.J., Yang T.Y., Chiang N.Y., Tsai W.P., Gordon S., Chang G.W., Kuo S.F., Lin H.H. (2018). High levels of soluble GPR56/ADGRG1 are associated with positive rheumatoid factor and elevated tumor necrosis factor in patients with rheumatoid arthritis. J. Microbiol. Immunol. Infect..

[61] Van Boekel M.A., Vossenaar E.R., van den Hoogen F.H., van Venrooij W.J. (2002). Autoantibody systems in rheumatoid arthritis: Specificity, sensitivity and diagnostic value. Arthritis Res..

[62] Vossenaar E.R., van Venrooij W.J. (2004). Anti-CCP antibodies, a highly specific marker for (early) rheumatoid arthritis. Clin. Appl. Immunol. Rev..

[63] Sidorov A., Beduleva L., Menshikov I., Terentiev A., Stolyarova E., Abisheva N. (2017). Fc fragments of immunoglobulin G are an inductor of regulatory rheumatoid factor and a promising therapeutic agent for rheumatic diseases. Int. J. Biol. Macromol..

[64] Aggarwal R., Liao K., Nair R., Ringold S., Costenbader K.H. (2009). Anti-citrullinated peptide antibody (ACPA) assays and their role in the diagnosis of rheumatoid arthritis. Arthritis Rheum..

[65] Manca M.L., Alunno A., D’Amato C., Bistoni O., Puxeddu I., Gerli R., Migliorini P., Pratesi F. (2017). Anti-citrullinated peptide antibodies profiling in established rheumatoid arthritis. Jt. Bone Spine.

[66] Derksen V.F.A.M., Huizinga T.W.J., van der Woude D. (2017). The role of autoantibodies in the pathophysiology of rheumatoid arthritis. Semin. Immunopathol..

[67] Yee A., Webb T., Seaman A., Infantino M., Meacci F., Manfredi M., Benucci M., Lakos G., Favalli E., Schioppo T. (2015). Anti-CarP antibodies as promising marker to measure joint damage and disease activity in patients with rheumatoid arthritis. Immunol. Res..

[68] Kumar S., Pangtey G., Gupta R., Rehan H.S., Gupta L.K. (2017). Assessment of anti-CarP antibodies, disease activity and quality of life in rheumatoid arthritis patients on conventional and biological disease-modifying antirheumatic drugs. Reumatologia.

[69] Shi J., van Steenbergen H.W., van Nies J.A.B., Levarht E.W.N., Huizinga T.W.J., van der Helm-van A.H.M., Toes R.E.M., Trouw L.A. (2015). The specificity of anti-carbamylated protein antibodies for rheumatoid arthritis in a setting of early arthritis. Arthritis Res. Ther..

[70] Wang X., Chen P., Cui J., Yang C., Du H. (2015). Keratin 8 is a novel autoantigen of rheumatoid arthritis. Biochem. Biophys. Res. Commun..

[71] Vossenaar E.R., Després N., Lapointe E., van der Heijden A., Lora M., Senshu T., van Venrooij W.J., Ménard H.A. (2004). Rheumatoid arthritis specific anti-Sa antibodies target citrullinated vimentin. Arthritis Res. Ther..

[72] Fadda S., Abolkheir E., Afifi R., Gamal M. (2016). Serum matrix metalloproteinase-3 in rheumatoid arthritis patients: Correlation with disease activity and joint destruction. Egypt. Rheumatol..

[73] Niki Y., Takeuchi T., Nakayama M., Nagasawa H., Kurasawa T., Yamada H., Toyama Y., Miyamoto T. (2012). Clinical significance of cartilage biomarkers for monitoring structural joint damage in rheumatoid arthritis patients treated with anti-TNF therapy. PLoS ONE.

[74] Paleolog E.M. (2009). The vasculature in rheumatoid arthritis: Cause or consequence?. Int. J. Exp. Pathol..

[75] Fardellone P., Séjourné A., Paccou J., Goëb V. (2014). Bone remodelling markers in rheumatoid arthritis. Mediat. Inflamm..

[76] Heo R., Park J.S., Jang H.J., Kim S.H., Shin J.M., Suh Y.D., Jeong J.H., Jo D.G., Park J.H. (2014). Hyaluronan nanoparticles bearing γ-secretase inhibitor: In vivo therapeutic effects on rheumatoid arthritis. J. Control. Release.

[77] Paiva K.B.S., Granjeiro J.M. (2017). Matrix metalloproteinases in bone resorption, remodelling, and repair. Prog. Mol. Biol. Transl. Sci..

[78] Darweesh H., Abbass D., Kadah R., Rashad A., Basel M.E., Nasr A.S. (2010). Serum and synovial cartilage oligomeric matrix protein (COMP) in patients with rheumatoid arthritis and osteoarthritis. Ind. J. Rheumatol..

[79] Lorenzo P., Aspberg A., Saxne T., Önnerfjord P. (2017). Quantification of cartilage oligomeric matrix protein (COMP) and a COMP neoepitope in synovial fluid of patients with different joint disorders by novel automated assays. Osteoarthr. Cartil..

[80] Gorman C., Leandro M., Isenberg D. (2003). B cell depletion in autoimmune disease. Arthritis Res. Ther..

[81] Choy E. (2003). Interleukin 6 receptor as a target for the treatment of rheumatoid arthritis. Ann. Rheum. Dis..

[82] Shaw T., Quan J., Totoritis M. (2003). B cell therapy for rheumatoid arthritis: The rituximab (anti-CD20) experience. Ann. Rheum. Dis..

[83] Taylor P.C. (2003). Anti-cytokines and cytokines in the treatment of rheumatoid arthritis. Curr. Pharm. Des..

[84] Burrage P.S., Mix K.S., Brinckerhoff C.E. (2006). Matrix metalloproteinases: Role in arthritis. Front Biosci..

[85] Albrecht K., Müller-Ladner U. (2010). Side effects and management of side effects of methotrexate in rheumatoid arthritis. Clin. Exp. Rheumatol..

[86] Guo Q., Wang Y., Xu D., Nossent J., Pavlos N.J., Xu J. (2018). Rheumatoid arthritis: Pathological mechanisms and modern pharmacologic therapies. Bone Res..

[87] Ma X., Xu S. (2013). TNF inhibitor therapy for rheumatoid arthritis. Biomed. Rep..

[88] Perdriger A. (2009). Infliximab in the treatment of rheumatoid arthritis. Biologics.

[89] Weinblatt M.E., Keystone E.C., Furst D.E., Moreland L.W., Weisman M.H., Birbara C.A., Teoh L.A., Fischkoff S.A., Chartash E.K. (2003). Adalimumab, a fully human anti-tumor necrosis factor alpha monoclonal antibody, for the treatment of rheumatoid arthritis in patients taking concomitant methotrexate: The ARMADA trial. Arthritis Rheum..

[90] Haraoui B., Bykerk V. (2007). Etanercept in the treatment of rheumatoid arthritis. Ther. Clin. Risk Manag..

[91] Mease P.J. (2011). Certolizumab pegol in the treatment of rheumatoid arthritis: A comprehensive review of its clinical efficacy and safety. Rheumatology (Oxford).

[92] Singh J.A., Noorbaloochi S., Singh G. (2010). Golimumab for rheumatoid arthritis: A systematic review. J. Rheumatol..

[93] Mok C.C. (2014). Rituximab for the treatment of rheumatoid arthritis: An update. Drug Des. Dev. Ther..

[94] Kaneko A. (2013). Tocilizumab in rheumatoid arthritis: Efficacy, safety and its place in therapy. Ther. Adv. Chronic Dis..

[95] Boyce E.G., Rogan E.L., Vyas D., Prasad N., Mai Y. (2018). Sarilumab: Review of a second IL-6 receptor antagonist indicated for the treatment of rheumatoid arthritis. Ann. Pharmacother..

[96] Ni H., Moe S., Myint K.T., Htet A. (2013). Oral janus kinase inhibitor for the treatment of rheumatoid arthritis: Tofacitinib. ISRN Rheumatol..

[97] Gras J. (2016). Baricitinib: JAK inhibition for rheumatoid arthritis. Drugs Today.

[98] Langdon K., Haleagrahara N. (2018). Regulatory T-cell dynamics with abatacept treatment in rheumatoid arthritis. Int. Rev. Immunol..

[99] Australian New Zealand Clinical Trials Registry Trial Review. https://www.anzctr.org.au/Trial/Registration/TrialReview.aspx?id=373425.

[100] NIH US National Library of Medicine Clinical Trials.gov. https://clinicaltrials.gov/ct2/show/NCT03410056.

[101] Crotti C., Raimondo M.G., Becciolini A., Biggioggero M., Favalli E.G. (2017). Spotlight on mavrilimumab for the treatment of rheumatoid arthritis: Evidence to date. Drug Des. Dev. Ther..

[102] Schiff M., Combe B., Dörner T., Kremer J.M., Huizinga T.W., Veenhuizen M., Gill A., Komocsar W., Berclaz P.Y., Ortmann R., Lee C. (2015). Efficacy and safety of tabalumab, an anti-BAFF monoclonal antibody, in patients with moderate-to-severe rheumatoid arthritis and inadequate response to TNF inhibitors: Results of a randomised, double-blind, placebo-controlled, phase 3 study. RMD Open.

[103] Huizinga T.W., Batalov A., Stoilov R., Lloyd E., Wagner T., Saurigny D., Souberbielle B., Esfandiari E. (2017). Phase 1b randomized, double-blind study of namilumab, an anti-granulocyte macrophage colony-stimulating factor monoclonal antibody, in mild-to-moderate rheumatoid arthritis. Arthritis Res. Ther..

[104] Cook A.D., Hamilton J.A. (2018). Investigational therapies targeting the granulocyte macrophage colony-stimulating factor receptor-α in rheumatoid arthritis: Focus on mavrilimumab. Ther. Adv. Musculoskelet. Dis..

[105] Kivitz A., Hazan L., Hoffman K., Wallin B.A. (2016). FRI0209 MORAb-022, an anti-granulocyte macrophage-colony stimulating factor (GM-CSF) monoclonal antibody (MAB): Results of the first study in patients with mild-to-moderate rheumatoid arthritis (RA). Ann. Rheum. Dis..

[106] Molfino N.A., Kuna P., Leff J.A., Oh C.K., Singh D., Chernow M., Sutton B., Yarranton G. (2016). Phase 2, randomised placebo-controlled trial to evaluate the efficacy and safety of an anti-GM-CSF antibody (KB003) in patients with inadequately controlled asthma. BMJ Open.

[107] Smolen J.S., Weinblatt M.E., van der Heijde D., Rigby W.F., van Vollenhoven R., Bingham C.O., Veenhuizen M., Gill A., Zhao F., Komocsar W.J. (2015). Efficacy and safety of tabalumab, an anti-B-cell-activating factor monoclonal antibody, in patients with rheumatoid arthritis who had an inadequate response to methotrexate therapy: Results from a phase III multicentre, randomised, double-blind study. Ann. Rheum. Dis..

[108] Mahtani K.R., Miller A., Rivero-Arias O., Heneghan C., Price C.P., Thompson M., Plüddemann A., Luqmani R. (2013). Autoimmune markers for the diagnosis of rheumatoid arthritis in primary care: Primary care diagnostic technology update. Br. J. Gen. Pract..

[109] Lilly Press Release Archives. http://lilly.mediaroom.com/index.php?s=9042&item=136985.

[110] Luime J.J., Colin E.M., Hazes J.M., Lubberts E. (2010). Does anti-mutated citrullinated vimentin have additional value as a serological marker in the diagnostic and prognostic investigation of patients with rheumatoid arthritis? A systematic review. Ann. Rheum. Dis..

[111] Maksymowych W.P., Naides S.J., Bykerk V., Siminovitch K.A., van Schaardenburg D., Boers M., Landewé R., van der Heijde D., Tak P.P., Genovese M.C., Weinblatt M.E. (2014). Serum 14-3-3η is a novel marker that complements current serological measurements to enhance detection of patients with rheumatoid arthritis. J. Rheumatol..

[112] Anderson J., Caplan L., Yazdany J., Robbins M.L., Neogi T., Michaud K., Saag K.G., O’Dell J.R., Kazi S. (2012). Rheumatoid arthritis disease activity measures: American college of rheumatology recommendations for use in clinical practice. Arthritis Care Res. (Hoboken).

[113] Study of KB003 in Biologics-Inadequate Rheumatoid Arthritis. https://clinicaltrials.gov/ct2/results?term=NCT00995449.

[114] Centola M., Cavet G., Shen Y., Ramanujan S., Knowlton N., Swan K.A., Turner M., Sutton C., Smith D.R., Haney D.J. (2013). Development of a multi-biomarker disease activity test for rheumatoid arthritis. PLoS ONE.

[115] Hambardzumyan K., Saevarsdottir S., Forslind K., Petersson I.F., Wallman J.K., Ernestam S., Bolce R.J., van Vollenhoven R.F. (2017). A multi-biomarker disease activity score and the choice of second-line therapy in early rheumatoid arthritis after methotrexate failure. Arthritis Rheumatol..

[116] Curtis J.R., Wright G.C., Strand V., Davis C.S., Hitraya E., Sasso E.H. (2017). Reanalysis of the multi-biomarker disease activity score for assessing disease activity in the AMPLEstudy: Comment on the article by Fleischmann et al. Arthritis Rheumatol..

[117] Chaves Chaparro L.M., Salvatierra Ossorio J., Raya Álvarez E. (2011). Predictors of response to biologic therapies in rheumatoid arthritis. Rheumatol. Clin..

[118] Lv Q., Yin Y., Li X., Shan G., Wu X., Liang D., Li Y., Zhang X. (2014). The status of rheumatoid factor and anti-cyclic citrullinated peptide antibody are not associated with the effect of anti-TNFα agent treatment in patients with rheumatoid arthritis: A meta-analysis. PLoS ONE.

[119] Fabris M., De Vita S., Blasone N., Visentini D., Pezzarini E., Pontarini E., Fabro C., Quartuccio L., Mazzolini S., Curcio F. (2010). Serum levels of anti-CCP antibodies, anti-MCV antibodies and RF IgA in the follow-up of patients with rheumatoid arthritis treated with rituximab. Autoimmun. Highlights.

[120] Marotta A., Maksymowych W. (2014). SAT0070 levels of 14-3-3Eta predict good EULAR response to anti-TNF treatment in patients with rheumatoid arthritis. Ann. Rheum. Dis..

[121] Morozzi G., Fabbroni M., Bellisai F., Cucini S., Simpatico A., Galeazzi M. (2007). Low serum level of COMP, a cartilage turnover marker, predicts rapid and high ACR70 response to adalimumab therapy in rheumatoid arthritis. Clin. Rheumatol..

[122] Choi I.Y., Gerlag D.M., Herenius M.J., Thurlings R.M., Wijbrandts C.A., Foell D., Vogl T., Roth J., Tak P.P., Holzinger D. (2015). MRP8/14 serum levels as a strong predictor of response to biological treatments in patients with rheumatoid arthritis. Ann. Rheum. Dis..

[123] Isgren A., Forslind K., Erlandsson M., Axelsson C., Andersson S., Lund A., Bokarewa M. (2012). High survivin levels predict poor clinical response to infliximab treatment in patients with rheumatoid arthritis. Semin. Arthritis Rheum..

[124] Benham H., Nel H.J., Law S.C., Mehdi A.M., Street S., Ramnoruth N., Pahau H., Lee B.T., Ng J., Brunck M.E. (2015). Citrullinated peptide dendritic cell immunotherapy in HLA risk genotype-positive rheumatoid arthritis patients. Sci. Transl. Med..

[125] Shabahang S., Li A.F., Escher A. (2010). Recent patents on immunoregulatory DNA vaccines for autoimmune diseases and allograft rejection. Recent Pat. DNA Gene Seq..

[126] Ratsimandresy R.A., Duvallet E., Assier E., Semerano L., Delavallée L., Bessis N., Zagury J.F., Boissier M.C. (2011). Active immunization against IL-23p19 improves experimental arthritis. Vaccine.

[127] Cohen S.B., Dore R.K., Lane N.E., Ory P.A., Peterfy C.G., Sharp J.T., van der Heijde D., Zhou L., Tsuji W., Newmark R., Denosumab Rheumatoid Arthritis Study Group (2008). Denosumab treatment effects on structural damage, bone mineral density, and bone turnover in rheumatoid arthritis: A twelve-month, multicenter, randomized, double-blind, placebo-controlled, phase II clinical trial. Arthritis Rheum..

[128] Takeuchi T., Tanaka Y., Ishiguro N., Yamanaka H., Yoneda T., Ohira T., Okubo N., Genant H.K., van der Heijde D. (2016). Effect of denosumab on Japanese patients with rheumatoid arthritis: A dose-response study of AMG 162 (Denosumab) in patients with rheumatoid arthritis on methotrexate to validate inhibitory effect on bone Erosion (DRIVE)-a 12-month, multicentre, randomised, double-blind, placebo-controlled, phase II clinical trial. Ann. Rheum. Dis..

[129] Feng G.D., Xue X.C., Gao M.L., Wang X.F., Shu Z., Mu N., Gao Y., Wang Z.L., Hao Q., Li W.N. (2014). Therapeutic effects of PADRE-BAFF autovaccine on rat adjuvant arthritis. BioMed Res. Int..

[130] Mould A.W., Scotney P., Greco S.A., Hayward N.K., Nash A., Kay G.F. (2008). Prophylactic but not therapeutic activity of a monoclonal antibody that neutralizes the binding of VEGF-B to VEGFR-1 in a murine collagen-induced arthritis model. Rheumatology (Oxford).

[131] Rosenthal K.S., Mikecz K., Steiner H.L., Glant T.T., Finnegan A., Carambula R.E., Zimmerman D.H. (2015). Rheumatoid arthritis vaccine therapies: Perspectives and lessons from therapeutic ligand epitope antigen presentation system vaccines for models of rheumatoid arthritis. Expert Rev. Vaccines.

[132] Chiu Y.G., Ritchlin C.T. (2017). Denosumab: Targeting the RANKL pathway to treat rheumatoid arthritis. Expert Opin Biol. Ther..

[133] Malemud C.J. (2015). Vaccine development for rheumatoid arthritis. Glob. Vaccines Immunol..

[134] Nandakumar K.S., Holmdahl R. (2005). Efficient promotion of collagen antibody induced arthritis (CAIA) using four monoclonal antibodies specific for the major epitopes recognized in both collagen induced arthritis and rheumatoid arthritis. J. Immunol. Methods.

[135] Hasselberg A., Schön K., Tarkowski A., Lycke N. (2009). Role of CTA1R7K-COL-DD as a novel therapeutic mucosal tolerance-inducing vector for treatment of collagen-induced arthritis. Arthritis Rheum..

[136] Zimmerman D.H., Taylor P., Bendele A., Carambula R., Duzant Y., Lowe V., O’Neill S.P., Talor E., Rosenthal K.S. (2010). CEL-2000: A therapeutic vaccine for rheumatoid arthritis arrests disease development and alters serum cytokine/chemokine patterns in the bovine collagen type II induced arthritis in the DBA mouse model. Int. Immunopharmacol..

[137] Dzhambazov B., Nandakumar K.S., Kihlberg J., Fugger L., Holmdahl R., Vestberg M. (2006). Therapeutic vaccination of active arthritis with a glycosylated collagen type II peptide in complex with MHC class II molecules. J. Immunol..

[138] Kochetkova I., Trunkle T., Callis G., Pascual D.W. (2008). Vaccination without autoantigen protects against collagen II-induced arthritis via immune deviation and regulatory T cells. J. Immunol..

[139] Luross J.A., Heaton T., Hirst T.R., Day M.J., Williams N.A. (2002). Escherichia coli heat-labile enterotoxin B subunit prevents autoimmune arthritis through induction of regulatory CD4+ T cells. Arthritis Rheum..

